# Supplementary material for: Populus euphratica JRL Mediates ABA Response, Ionic and ROS Homeostasis in Arabidopsis under Salt Stress
Source: Int J Mol Sci. 2019 Feb 14;20(4):815. doi: 10.3390/ijms20040815 (PMC6412788; doi:10.3390/ijms20040815)
Supplement: Supplementary file 1 [file ijms-20-00815-s001.pdf]

Supplementary Table S1 Gene-specific primer sequences for real-time quantitative PCR assays and full-length cloning of *PeJRL*.

Gene Name	Forward primer (5′ to 3′)	Reverse primer (5′ to 3′)	Accession number
AtACTIN2-RT	GGTAACATTGTGCTCAGTGGTGG	AACGACCTTAATCTTCATGCTGC	AT3G18780
AtSOD-RT	AGGAAACATCACTGTTGGAGAT	GAGTTTGGTCCAGTAAGAGGAA	AT1G08830
AtPOD-RT	CGTGCCCTTCATATTGTTGG	GACGCCATCAACAACGAGTC	AT1G48130
AtCAT-RT	AGGATCAAACTTTGAGGGGTAG	CTTGTGGTTCCTGGAATCTACT	AT1G20620
AtHKT1-RT	GATTTGTCCCCACGAATGAGA	CAAAACCAAGAAGCAAGGGAAC	AT4G10310
AtSOS1-RT	GTGAAGCAATCAAGCGGAAA	TGCGAAGAAGGCGTAGAACA	AT2G01980
AtAHA1-RT	CACAAACATTTACCGAAAACCA	CAAATTTGCAAAGCTCATATCG	AT2G18960
AtAHA2-RT	TGACTGATCTTCGATCCTCTCA	GAGAATGTGCATGTGCCAAA	AT4G30190
AtRBOHD-RT	AGCTTCACAATTATTGCACGAG	TCTCCAGTTAGGTTTAGCGAAG	AT5G47910
AtRBOHF-RT	TATTGGAGACCATCTTGCTTGT	CGTTAAAACCGGTTAGTCGATC	AT1G64060
AtNCED2-RT	GATTCCTGATCAACAAGTCGTG	GAAACCTTTTCTCCGTCGAAAA	AT4G18350
AtNCED9-RT	GCGGGCTATTTGGGTTAGTC	CGGTAAATCGTCTTCGGACA	AT1G78390
ABF4-RT	AACAACTTAGGAGGTGGTGGTC	CTTCAGGAGTTCATCCATGTTC	AT3G19290
DREB1A-RT	GATCAGCCTGTCTCAATTTC	CTTCTGCCATATTAGCCAAC	AT4G25480
DREB2A-RT	AAGGTAAAGGAGGACCAGAG	ACACAACCAGGAGTCTCAAC	AT5G05410
MYB2-RT	TGCTCGTTGGAACCACATCG	ACCACCTATTGCCCCAAAGAGA	AT2G47190
RAB18-RT	CAGCAGCAGTATGACGAGTA	CAGTTCCAAAGCCTTCAGTC	AT5G66400
RD29A-RT	ATCACTTGGCTCCACTGTTGTTC	ACAAAACACACATAAACATCCAAAGT	AT5G52310
SnRK2.2-RT	ATATGCCATCGGGATCTGAA	TTGGTTGGGAATGAAGAACAG	AT3G50500

SnRK2.3-RT	GTTGGATGGAAGTCCT GCTC	TGCCATCATATTCCTG ACGA	AT5G66880
ABI5-RT	CAATAAGAGAGGGATA GCGAACGAG	CGTCCATTGCTGTCTC CTCCA	AT2G36270
PeJRL-RT	CACATGGAAGCATTTC AGTTGG	TCACCACCATTCCAG CATAATTTCC	XM_011003496. 1
PeJRL	ATGGCATCCTTGGAAC GAATC	TTAGATTGTCGTCTCT GGTTTGAC	XM_011003496. 1
PeACTIN7-RT	ATTGGCCTTGGGGTTAA GAG	CACACTGGAGTGATG GTTGG	XM_011034907
